# Restoring Ambiguous Boundaries: An Efficient and Robust Framework for Underwater Camouflaged Object Detection

**DOI:** 10.3390/s26030872

**Published:** 2026-01-28

**Authors:** Zihan Wei, Yucheng Zheng, Yaohua Shen, Xiaofei Yang

**Affiliations:** Automation College, Jiangsu University of Science and Technology, Zhenjiang 212100, China; 232210307107@stu.just.edu.cn (Z.W.); yxfei_0809@just.edu.cn (X.Y.)

**Keywords:** underwater camouflaged object, boundary ambiguity, frequency-domain analysis, lightweight object detection, robustness in degraded environments

## Abstract

The efficacy of Underwater Camouflaged Object Detection (UCOD) is fundamentally constrained by severe boundary ambiguity, where biological mimicry blends targets into complex backgrounds and aquatic optical degradation erodes edge details. We propose a lightweight boundary perception detector named CAR-YOLO (Camouflage Ambiguity Resolution YOLO). Specifically, a frequency-domain dual-path mechanism (FRM-DWT/EG-IWT) leverages selective wavelet aggregation and dynamic injection to recover high-frequency edges. Subsequently, these high-frequency cues are synergized with low-frequency semantic information via the Low-level Adaptive Fusion (LAF) module. To further address noisy samples, an Uncertainty Calibration Head (UCH) refines supervision via prediction consistency. Finally, we constructed specialized datasets based on public data for training and evaluation, including UCOD10K and UWB-COT220. On UCOD10K, CAR-YOLO achieves 27.1% mAP50–95, surpassing several state-of-the-art (SOTA) methods while reducing parameters from 2.58 M to 2.43 M and GFLOPs from 6.3 to 5.9. On the challenging UWB-COT220 benchmark, the model attains 30.7% mAP50–95, marking a 7.7-point improvement over YOLOv11. Furthermore, cross-domain experiments on UODD demonstrate strong generalization. These results indicate that CAR-YOLO effectively mitigates boundary ambiguity, achieving an optimal balance between accuracy, robustness, and efficiency.

## 1. Introduction

Underwater Camouflaged Object Detection (UCOD) [1,2] is a challenging task distinct from both general Underwater Object Detection (UOD) [3] and terrestrial Camouflaged Object Detection (COD) [4]. General UOD typically targets distinct objects like divers or shipwrecks that exhibit strong contrast against the water column. In contrast, UCOD targets rely on biological camouflage to mimic surrounding textures and patterns. This significantly limits the effectiveness of standard feature extraction. Terrestrial COD tasks typically operate in clear air, benefiting from high visibility and stable transmission. Conversely, the underwater environment acts as an optical filter. This causes severe degradation like light attenuation, scattering, and color casts. Therefore, the unique difficulty of UCOD stems from the interaction of these two factors. Intrinsic camouflage reduces the contrast between the target and background, while extrinsic water turbidity erodes the high-frequency edge information essential for breaking that camouflage. This combination causes ambiguity in target boundaries, making it challenging to distinguish targets from background noise. Autonomous platforms, such as Remotely Operated Vehicles (ROVs) [5] and Unmanned Surface Vehicles (USVs) [6,7], are pivotal for marine ecological protection and resource exploration. These missions critically depend on robust, real-time perception. However, the inherent complexity of UCOD poses a severe challenge to this require.

Existing SOTA object detectors are structurally inadequate for this specific problem. While heavyweight architectures are computationally prohibitive for real-time deployment on such platforms, lightweight object detectors like the YOLO family [8] are favored for their efficiency. However, these models were originally designed for high-contrast terrestrial objects. Consequently, their standard architectures struggle with severe high-frequency information loss and lack robustness against the low signal-to-noise ratios typical of UCOD.

To address these challenges and meet practical survey demands, we adopt YOLOv11 as the baseline. Two core architectural weaknesses in UCOD have been systematically examined. First, traditional models suffer from severe high-frequency information loss. Second, they struggle to process features with low signal-to-noise ratios. The main contributions of this work are as follows:

(1) A framework, CAR-YOLO, is proposed to address boundary ambiguity in UCOD. The proposed framework incorporates three core modules. First, frequency-domain dual-path mechanisms (FRM-DWT and EG-IWT) employ selective wavelet transforms to disentangle and restore high-frequency edge details. Second, the LAF dynamic fusion module balances high- and low-frequency features to enhance contextual reasoning. Third, the UCH adopts a refined supervision strategy by calibrating synergistic loss functions, thereby ensuring robust supervision of ambiguous signals.

(2) CAR-YOLO achieves high efficiency through a lightweight design. The LAF module replaces expensive 1 × 1 convolutions with a low-rank–inspired structure to minimize computational cost. Additionally, the UCH functions as a strict supervision strategy, incurring zero overhead during inference. Collectively, these optimizations ensure CAR-YOLO is lighter and faster than the baseline without sacrificing accuracy.

(3) To address the scarcity of specialized datasets in UCOD, we constructed and benchmarked UCOD10K and UWB-COT220 to facilitate future research. Extensive experiments on these benchmarks demonstrate the superiority of CAR-YOLO. It consistently surpasses baseline and SOTA methods, reconciling the demands of accuracy, robustness, and real-time efficiency.

## 2. Related Work

Existing SOTA methods generally follow two directions to address intrinsic camouflage and extrinsic degradation. The first stream involves complex segmentation architectures. Transformer-based COD models, such as FSPNet [9], enhance global context and boundary detection. However, tailored for in-air camouflage, it overlooks realistic underwater color shifts and contrast loss. Moreover, it lacks mechanisms to recover high-frequency boundaries eroded by scattering. In a similar vein, MAMIFNet [10] integrates frequency and spatial information via parallel branches. Yet, its heavy design targets general benchmarks rather than underwater degradation. This high computational cost hinders real-time deployment. Meanwhile, ChirpFAN [11] models non-stationary frequency modulations for remote sensing but lacks the specific boundary restoration needed for UCOD.

The second stream focuses on lightweight detection frameworks. SAM-based approaches improve segmentation quality but incur prohibitive computational costs. Dual-SAM [12], for example, integrates underwater priors yet retains the heavy ViT backbone. It focuses on mask segmentation rather than the lightweight box-level detection required here. HQ-SAM [13] employs extra layers to sharpen masks, inevitably increasing inference overhead. Lacking uncertainty modeling, this model treats ambiguous boundaries as certain. VL-SAM2 [14] introduces language and motion guidance to SAM2. This reliance on text or video inputs makes it unsuitable for single-frame UCOD tasks. DINO [15] achieves anchor denoising but imposes excessive computational burdens on edge devices for USVs or ROVs [5,6,7]. Its architecture is also ill-suited for the low-contrast boundaries inherent to camouflage.

Second, lightweight object detectors are widely adopted for general UOD. While effective, they mainly address image-level degradation rather than intrinsic camouflage and boundary ambiguity. For instance, BG-YOLO [16] employs a bidirectional-guided framework to fuse enhancement and detection features. However, it operates only in the spatial domain, failing to preserve explicitly camouflaged high-frequency edges. Similarly, TC-YOLO [17] integrates Coordinate Attention and Swin Transformer blocks to mitigate noise. Yet, its design lacks frequency-aware modeling, limiting its ability to decouple targets from texture-similar backgrounds.

Joint restoration–detection networks, such as Yeh et al. [18], improve global low-frequency appearance using a color-conversion subnetwork. However, this process can smooth away fragile boundary textures that are crucial for UCOD. Channel-sharpening attention for underwater species detection [19] reweights attenuation-sensitive channels, but it does not explicitly address intrinsic camouflage or boundary uncertainty. Small-object variants such as LFN-YOLO [20] and Dynamic YOLO [21] reduce parameter number via lightweight convolutions. Nevertheless, they still rely on standard 1 × 1 convolutions and deterministic losses, lacking explicit restoration of high-frequency boundaries or uncertainty-aware supervision for ambiguous regions.

While the aforementioned methods are effective, this standard setup overlooks the frequency loss and misalignment issues typical in underwater scenes. Overall, current COD and UOD models either sacrifice efficiency or overlook frequency-aware modeling and boundary ambiguity. These limitations motivate the need for a lightweight, frequency-aware, context-adaptive, and uncertainty-calibrated detector for UCOD.

Considering these factors, we select YOLOv11 [22] as our baseline for its strong balance of speed and accuracy. Such frameworks have proven effective in handling complex terrestrial scenarios, as demonstrated by recent datasets like RSUD20K [23], which focuses on road scene understanding for autonomous driving. The proposed method first processes images through a C3k2 backbone to extract features. These features are then merged by a PANet neck, ensuring that objects of different sizes are captured. Finally, a decoupled head generates the output by handling classification and positioning tasks separately. Experiments verify that our improved model performs reliably, achieving high detection confidence in underwater camouflage tasks.

## 3. Method

### 3.1. Overall Network Architecture

**Figure 1:**
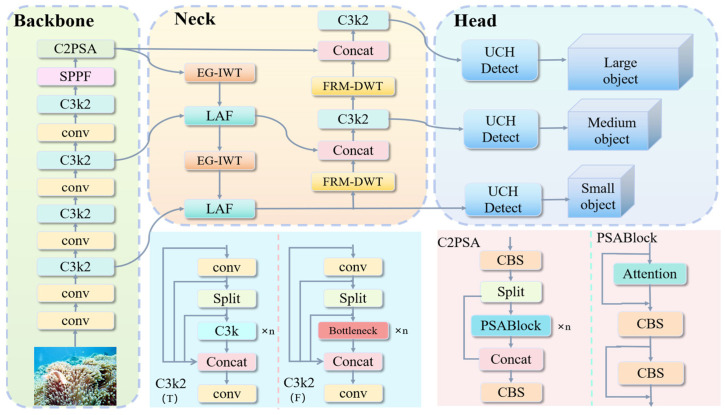
Overall Architecture of CAR-YOLO.

In this paper, we propose a new architecture named CAR-YOLO to address the limitations of YOLOv11 for UCOD. As illustrated in Figure 1, CAR-YOLO retains the three-stage design of YOLOv11: backbone, neck, and detection head. We systematically replace the sampling and fusion components responsible for boundary information loss. Specifically:

1. In the neck, we substitute all standard downsampling convolutions (stride = 2) and interpolation-based upsampling operators with FRM-DWT and EG-IWT blocks. This integration establishes frequency-domain paths that explicitly preserve high-frequency boundary details.
2. At each scale-interaction node in the neck, we replace the original C3k2 fusion modules with LAF blocks. It enables the model to perform low-rank attention fusion between top–down and bottom–up feature maps.
3. Finally, we substitute the original detection head with the UCH. This module refines supervision by integrating prediction consistency, uncertainty-aware regression, Inner-IoU sample matching, and QFL. These components synergize to mitigate the severe boundary ambiguity of camouflaged targets.

### 3.2. Frequency-Domain Boundary Preservation and Restoration

The standard YOLOv11 architecture relies on frequency-agnostic operators where the strided convolution functions as a low-pass filter, causing the irreversible loss of high-frequency details. Similarly, standard bilinear interpolation yields only blurry approximations and fails to restore information critical for UCOD.

To address this limitation, we introduce the Discrete Wavelet Transform and Inverse Discrete Wavelet Transform to design a matched set of frequency-domain sampling modules. Specifically, we propose FRM-DWT for downsampling to explicitly preserve boundary details and EG-IWT for upsampling to effectively restore them. Unlike existing frequency-aware models that typically treat frequency analysis as an auxiliary reconstruction task where cues are extracted via parallel branches [24], we embed frequency awareness directly into the core sampling structure of the detector. Furthermore, acknowledging that underwater high-frequency signals often contain suspended particle noise rather than valid textures, we integrate a lightweight sub-band soft-selection mechanism. This design allows the model to adaptively filter out noise while strictly preserving reliable edge details during feature resampling.

#### 3.2.1. FRM-DWT Module Structure

**Figure 2:**
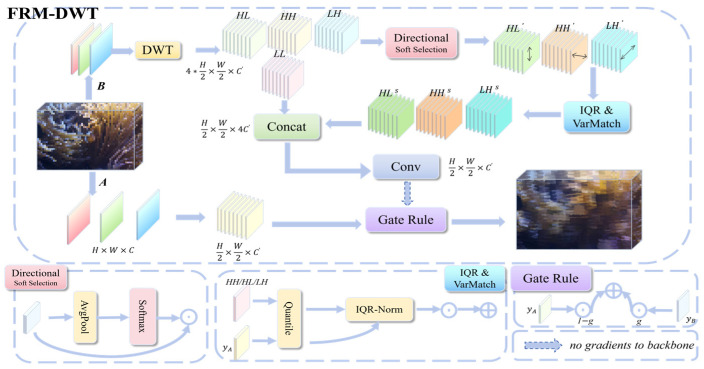
FRM-DWT Module Architecture.

The overall architecture of the FRM-DWT module is illustrated in Figure 2. It employs a dual-path system to regulate feature flow. The Semantic Path (path A) retains original features to ensure gradient stability. This design preserves features learned by the pretrained backbone, ensuring stable initialization and essential semantic representation during early training. In parallel, the Frequency Path (path B) utilizes a Discrete Wavelet Transform (DWT) to decompose the input, and independently processes high-frequency boundary details before re-injection. It yields one low-frequency component (LL) and three high-frequency sub-bands (LH, HL, HH). The LL component approximates the original feature, retaining main object shapes and lighting variations. The sub-bands (LH, HL, HH) capture horizontal, vertical, and diagonal edge details, respectively. This strategic separation avoids the gradient contamination often caused by straightforward DWT integration. Standard DWT extracts high-frequency information by decomposing spatial signals. However, directly mixing these raw frequency signals with spatial features pollutes the main gradient flow. This interference often leads to training instability.

To refine these details, Path B integrates a Directional Soft-Selection mechanism to enhance critical edge structures while suppressing noise. Specifically, it calculates the signal strength of each high-frequency sub-band via Global Average Pooling:

(1) $$E_{LH}=\mathrm{AvgPool}\left(\left|LH\right|\right),\;E_{HL}=\mathrm{AvgPool}\left(\left|HL\right|\right),\;E_{HH}=\mathrm{AvgPool}\left(\left|HH\right|\right)$$

Subsequently, a Softmax function converts these energy values into importance weights:


(2) $$\omega =\mathrm{softmax}\left(\frac{1}{\tau }\left[E_{LH},E_{HL},E_{HH}\right]\right)$$

These weights are then applied to the respective sub-bands. This modulation amplifies significant boundaries while suppressing background clutter:


(3) $$LH^{\prime }=\omega_{LH}⊙LH,\;HL^{\prime }=\omega_{HL}⊙HL,\;HH^{\prime }=\omega_{HH}⊙HH$$

To ensure statistical validity across different scales, a robust normalization strategy is employed:

(4) $$S=\left[LH^{\prime },HL^{\prime },HH^{\prime }\right]$$

(5) $$\mathrm{IQR}(S)=Q_{3}(S)-Q_{1}(S)$$

where $Q_{1}\left(\cdot \right)$ and $Q_{3}\left(\cdot \right)$ denote the 25th and 75th percentiles computed per channel over spatial locations.

(6) $$\mathrm{IQRNorm}(S)=\frac{S-\mathrm{median}(S)}{\mathrm{IQR}(S)+\epsilon }$$

(7) $$\hat{S}=\mathrm{IQRNorm}(S)$$

(8) $$S^{\prime }=\hat{S}\cdot {\mathrm{IQR}}_{ref}+{\mathrm{median}}_{ref}$$

(9) $$S^{\prime }=\left[LH^{s},HL^{s},HH^{s}\right]$$

Specifically, ${\mathrm{median}}_{\mathrm{ref}}$ and ${\mathrm{IQR}}_{\mathrm{ref}}$ are the reference median and interquartile range, derived spatially from the semantic feature $Y_{A}$ on a per-channel basis.

The processed high-frequency sub-bands are concatenated with the LL component to produce the Path B output $Y_{B}$, as shown in Figure 2. This output is finally fused with the Path A output $Y_{A}$ using a gradient-free gate  g. This gate dynamically adjusts the weights based on edge energy:

(10) $$Y=(1-g)\cdot Y_{A}+g\cdot Y_{B}$$

Crucially, the gate g is detached from backpropagation. This isolation prevents gradients from Path B from interfering with Path A. Consequently, the module maintains stable feature learning while explicitly restoring high-frequency boundary details typically lost during standard downsampling. This approach prevents gradient pollution while recovering the boundary details typically lost during downsampling. By decoupling frequency and spatial signals, the module ensures stable training without sacrificing the fine-grained features needed for accurate detection.

Regarding deployment, the FRM-DWT module is strategically activated only at the last two down-sampling stages. Deep layers in the YOLOv11 backbone extract highly semantic features but suffer from the loss of fine boundary cues. Integrating FRM-DWT at these stages restores critical high-frequency guidance for precise localization. This configuration minimizes computational overhead, achieving an optimal balance between accuracy and efficiency.

#### 3.2.2. EG-IWT Module Structure

**Figure 3:**
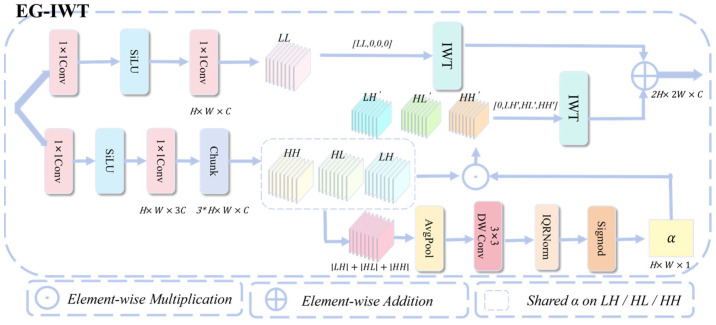
EG-IWT Module Architecture.

Integrated into the feature-pyramid fusion stage, the EG-IWT module utilizes a dynamic, gradient-free guidance map G to adaptively regulate high-frequency detail injection Figure 3. This design effectively mitigates limitations inherent in existing approaches. Standard YOLOv11 upsampling frequently yields block artifacts and blurred boundaries. Meanwhile, traditional Inverse Wavelet Transform (IWT) methods rely on a static scalar γ. The fixed value tends to amplify noise in smooth areas while failing to sharpen crucial edges sufficiently.

To enhance boundaries effectively, the local edge energy map E is derived by aggregating high-frequency details. We implement this aggregation with channel-wise averaging, taking the mean over C to produce a single-channel energy response.

(11) $$E={\mathrm{Conv}}_{3\times 3}^{dw}\left({\mathrm{AvgPool}}_{c}\left(\left|LH\right|+\left|HL\right|+\left|HH\right|\right)\right)$$

This energy E is then normalized via IQRNorm and passed through a Sigmoid activation to create the dynamic guidance map G:

(12) $$G=\sigma \left(\mathrm{IQRNorm}(E)\right)$$

As illustrated in Figure 3, the map is applied to the stacked high-frequency sub-bands by element-wise multiplication. This operation amplifies the signal in edge regions while suppressing background noise:

(13) $$LH^{\prime }=G⊙LH,\;HL^{\prime }=G⊙HL,\;HH^{\prime }=G⊙HH$$

Finally, an inverse Haar transform yields the reconstructed high-resolution feature map:

(14) $$Y=\mathrm{IWT}\left(LL,0,0,0\right)+\mathrm{IWT}\left(0,LH^{\prime },HL^{\prime },HH^{\prime }\right)$$

This design allows EG-IWT to precisely restore boundaries by strongly injecting high-frequency details along edges while suppressing noise in flat regions.

### 3.3. LAF Low-Rank Dynamic Fusion Module

**Figure 4:**
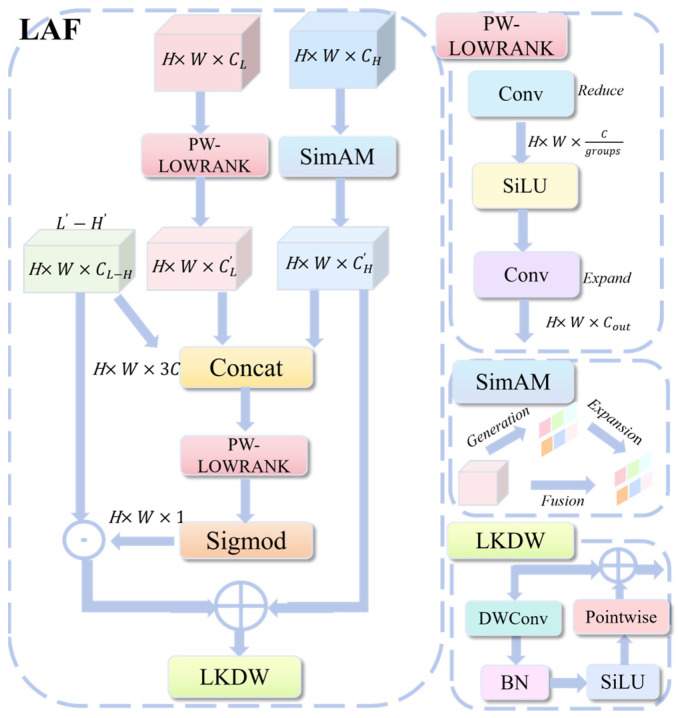
LAF Module Architecture.

The LAF module, illustrated in Figure 4, integrates the restored high-frequency details with high-level semantics. This design addresses the limitations of the standard C3k2 module used in YOLOv11. The standard approach relies on simple concatenation for cross-layer fusion. While efficient for general detection, it fails to balance features in the UCOD task. Specifically, it cannot effectively manage the conflict between strong semantic information and the fragile boundaries characteristic of camouflaged targets.

By incorporating a learnable scaling factor β, LAF achieves dynamic fusion of high and low-frequency information.

(15) $$F_{out}=\left(1-\beta M\right)⊙F_{H}+\left(\beta M\right)⊙F_{L}$$

To generate the weight M, the module integrates low-level features, high-level features, and their difference. This combined information is processed to capture spatial importance, followed by Sigmoid normalization.

(16) $$M=\sigma \left(\mathrm{Conv}\left(\mathrm{Concat}\left(F_{L},F_{H},F_{L}-F_{H}\right)\right)\right)$$

However, implementing this strategy with standard 1 × 1 Conv introduces significant parameter overhead. To address this, we adopt the PW-LOWRANK module, inspired by Low-Rank Adaptation [25], to reconstruct the key projection and weight computation layers. By using low-rank decomposition and group convolution, PW-LOWRANK requires far fewer parameters than standard convolutions. This substitution makes the LAF architecture highly efficient.

Meanwhile, to ensure the dynamic weight M is calculated from reliable information, we introduce the parameter-free SimAM attention mechanism [26]. It pre-refines the high-level features. Noise will be blended with the high-level features, as UCOD images often suffer from low signal-to-noise ratios. SimAM adaptively suppresses these irrelevant responses and highlights the semantic regions that are truly important for fusion.

Finally, we employ the Large-Kernel Depth-Wise convolution (LKDW) [27] to process the fused feature $F_{out}$. This parameter-efficient operation significantly expands the module’s effective receptive field. It enables the model to better recognize the spatial context, which is crucial for distinguishing large-area camouflage textures. By interpreting fine details within a broader context, the LAF module improves its ability to identify camouflage patterns.

### 3.4. Prior-Guided Detection Head and Loss Functions

Next, we introduce the UCH scheme as a specialized supervision framework for the training phase. Underwater scenarios are plagued by inherent boundary ambiguity and severe annotation noise, yet standard YOLO detection heads process samples indiscriminately. Recent research indicates that such uniform treatment neglects the inherent inequality of bounding box distributions [28]. Furthermore, the lack of gradient calibration often leads to misalignment between regression loss and detection quality [29]. This limitation yields suboptimal guidance and degrades the quality of the training process due to noisy supervision.

This training-only framework coordinates four mechanisms to calibrate the learning process. First, we use Inner-IoU [30] to generate strong gradients, ensuring effective learning even for samples with low overlap. To prevent the model from overfitting to noise within these gradients, we integrate Uncertainty-Aware Regression. This acts as a dynamic filter that specifically down-weights ambiguous boundaries in the loss function. Simultaneously, a Prediction Consistency Strategy is used to focus classification on stable predictions. Finally, a Ranking-Based Consistency Loss bridges the two branches, ensuring that the most accurate detections receive the highest confidence scores. Crucially, all these components are discarded during inference to maintain zero overhead.

We start by describing the geometric foundation of the UCH framework. In cases where predictions exhibit little to no overlap with the ground truth, standard IoU-based losses such as GIoU [31] yield negligible gradients, severely hindering model optimization. To address this gradient vanishing issue, we incorporate Inner-IoU [30] as the regression baseline. By introducing an auxiliary bounding box, this method generates stronger gradient signals, ensuring effective learning even under low-overlap conditions.

However, strong gradients alone can be detrimental if the annotations themselves contain structural noise. To prevent the model from overfitting to such ambiguous signals, we augment the regression branch with Uncertainty-Aware Regression. In addition to the standard bounding box coordinates, the regression head predicts a four-dimensional uncertainty vector $\hat{s_{i}}$ This vector is integrated into the final regression loss $L_{\mathrm{UAReg}}$, which is formulated as:

(17) $$L_{\mathrm{UAReg}}=\exp(-{\bar{s}}_{i})\cdot L_{\mathrm{Inner}}+{\bar{s}}_{i}$$

where $L_{\mathrm{Inner}}$ is Inner-IoU Regression Loss, to aggregate the dimensional uncertainties into a unified metric, ${\bar{s}}_{i}$ is computed as the mean of the four-dimensional vector $\hat{s_{i}}$:

(18) $${\bar{s}}_{i}=\frac{1}{4}\sum_{j=1}^{4}{\hat{s}}_{i,j}$$

This probabilistic loss dynamically reduces the weight of noisy or highly ambiguous boxes. Crucially, it still provides informative gradients in the low-IoU regime.

Simultaneously, on the classification branch, we employ a Prediction Consistency Strategy to filter out noise. We derive a reliability weight $r_{i}$ by jointly assessing the classification confidence and localization accuracy. a high $r_{i}$ is assigned only when a prediction concurrently yields a high classification score and a high IoU overlap.

Building on this reliability metric, we address the misalignment between detection confidence and localization quality using Quality Focal Loss (QFL) [32]. The classification target is redefined from a discrete label {0,1} to the continuous IoU score, forcing the classification score to strictly reflect localization accuracy. To integrate the consistency guidance, we scale this loss using the reliability weight $r_{i}\cdot L_{\mathrm{QFL}}$. This formulation dynamically focuses the model’s learning capacity on stable, high-quality predictions.

**Figure 5:**
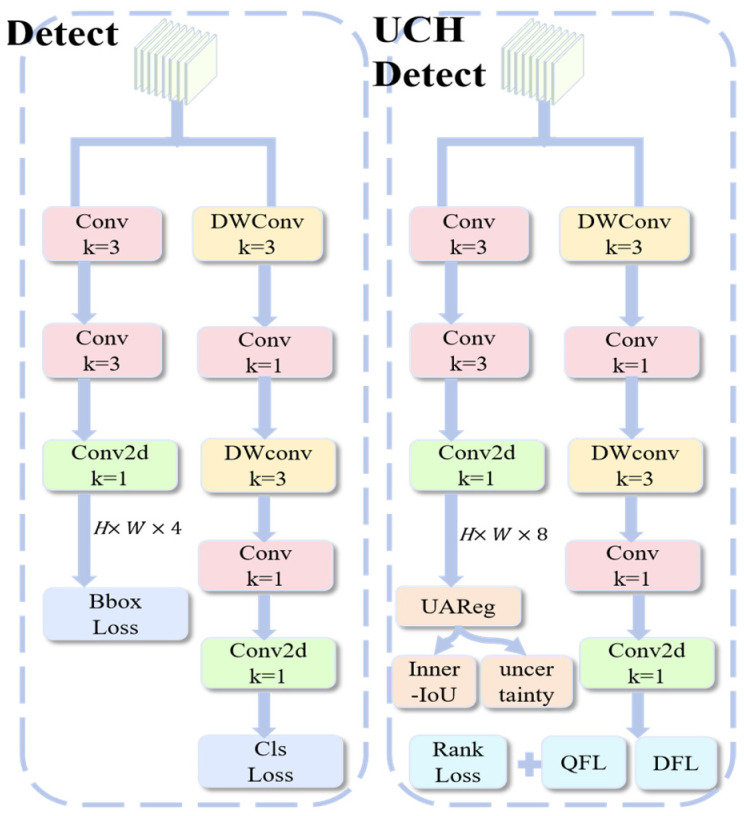
UCH Module Architecture.

Finally, to synchronize the regression and classification branches, we apply a Ranking-Based Consistency Loss $L_{\mathrm{Rank}}$. In standard object detectors, the classification branch focuses solely on category distinction, often assigning high confidence to poorly localized boxes. To correct this, $L_{\mathrm{Rank}}$ enforces a strict constraint where the classification score must jointly represent both category probability and localization quality. Specifically, it forces the model to assign higher scores only to predictions that have higher IoU with the ground truth. This ensures that during inference, the most precise detections naturally rise to the top of the ranking list. These mechanisms only modify the training objectives and sample assignment, leaving the inference pipeline unchanged. Thus, they introduce zero additional runtime overhead. By combining the joint supervision of $L_{\mathrm{QFL}}$ and $L_{\mathrm{Inner}}$ with the calibration mechanisms for uncertainty and consistency introduced by UCH, the proposed model effectively addresses boundary ambiguity and structural noise in UCOD. The overall architecture of the UCH head and its associated losses is illustrated in Figure 5.

### 3.5. Final Loss Function

To unify the synergistic mechanisms provided by the UCH scheme, the total training loss is designed as a weighted sum of classification, regression, and ranking components. Incorporating the reliability-weighted QFL and the uncertainty-aware Inner-IoU, the final objective function is formulated as follows:

(19) $$L_{\mathrm{total}}=\frac{1}{N_{\mathrm{pos}}}\sum_{i\in \mathrm{Pos}}(\lambda_{\mathrm{cls}}r_{i}L_{\mathrm{QFL}}(p_{i},q_{\mathrm{iou},i})+\lambda_{\mathrm{rank}}L_{\mathrm{Rank},i}+\lambda_{\mathrm{box}}L_{\mathrm{UAReg}}(p_{\mathrm{pred},i},b_{\mathrm{gt},i},{\hat{s}}_{i})+\lambda_{\mathrm{dfl}}L_{\mathrm{DFL},i})$$

where $N_{pos}$ denotes the number of positive samples, and $L_{\mathrm{DFL}}$ represents the standard Distribution Focal Loss. The hyperparameters $\lambda_{\mathrm{cls}}$, $\lambda_{\mathrm{rank}}$, $\lambda_{\mathrm{box}}$, and $\lambda_{\mathrm{dfl}}$ are employed to balance the contribution of each task.

The efficacy of this function stems from the distinct roles of its calibration components. Specifically, $L_{\mathrm{UAReg}}$ serves as the geometric foundation. It utilizes the predicted uncertainty vector $\hat{s_{i}}$ to dynamically down-weight the Inner-IoU loss for ambiguous boundaries. In this way, it acts effectively as a learned noise filter. Simultaneously, the reliability weight $r_{i}$ is derived from the Prediction Consistency Strategy. This weight quantifies the alignment between classification confidence and localization accuracy. By scaling $L_{\mathrm{QFL}}$, it focuses the model’s learning capacity on structurally stable predictions. Furthermore, $L_{\mathrm{Rank}}$ enforces that classification scores must remain proportional to IoU overlap. This constraint directly penalizes high-confidence predictions that lack precise localization.

In summary, the framework acts as a dynamic filter during training. It suppresses noise from ambiguous boundaries and aligns confidence scores with localization quality, ensuring the model focuses on reliable predictions.

## 4. Experiments and Results

In order to comprehensively evaluate our method, we design a series of experiments. The datasets, evaluation metrics, and specific implementation details are first given to ensure reproducibility. Through extensive ablation studies and robustness analyses, we systematically validate the actual contributions of each innovative component proposed, including FRM-DWT, EG-IWT, LAF, and the UCH implicit supervision calibration strategy.

### 4.1. Experimental Design

#### 4.1.1. Experimental Environment and Implementation Details

Experiments were conducted on Windows 11, using Python V3.12.11 and PyTorch 2.9.0 (CUDA 12.8), implementing YOLOv11 based on Ultralytics 8.3.223. The hardware included a server equipped with an NVIDIA GeForce RTX 4090 GPU and a laptop with an NVIDIA GeForce RTX 4060. The model training parameters are shown in Table 1.

The hyperparameters were determined based on preliminary empirical analysis of model convergence. To ensure a fair comparison, they were kept consistent across all experiments. The sole exception was the batch size for the UWB-COT220 dataset, which was adjusted to 16 to optimize training efficiency. Multiple data augmentation methods were adopted to improve robustness and generalization ability, including HSV color space transformation, random cropping, translation, horizontal flipping, and mosaic augmentation.

#### 4.1.2. Dataset Splitting

UCOD lacks specialized benchmarks, compelling reliance on general datasets. Even with official bounding boxes, the rectangular format introduces structural noise. Enclosed background pixels visually mimic the camouflaged target, causing severe semantic ambiguity distinct from standard detection. Mainstream camouflaged object detection models are built on cross-domain datasets, which leads to poor generalization in a complex underwater environment. Moreover, traditional UOD methods scarcely consider biological camouflage, where organisms are heavily mixed with the background.

**Figure 6:**
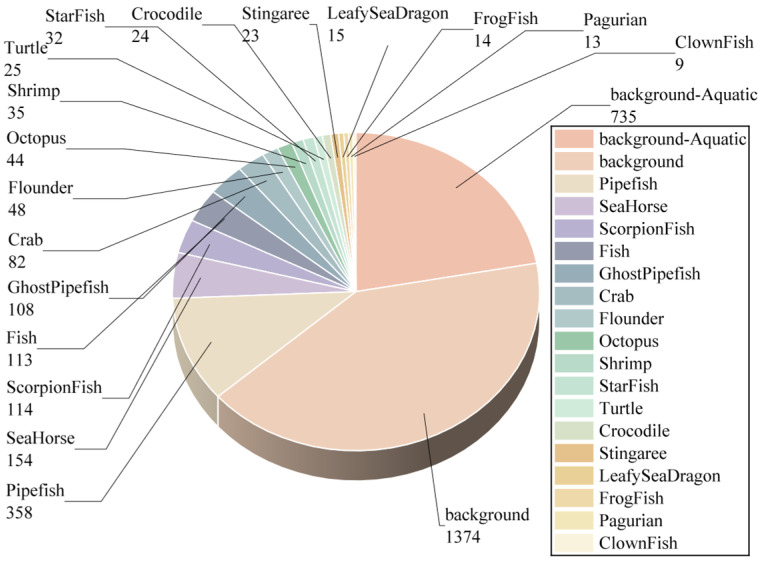
Category Distribution of the UCOD10K Dataset.

To address this gap, we constructed the UCOD10K dataset as our core benchmark, supplemented by UWB-COT220. This dataset comprises 3320 images curated from the authoritative COD10K benchmark [1], specifically retaining all underwater scenes. We employed Python scripts to extract aquatic scenes based on original file naming conventions. Subsequently, manual verification was performed to ensure each image represents a genuine underwater environment with valid targets. We normalized the original bounding box annotations to the YOLO format while simultaneously correcting artifacts such as out-of-bound coordinates, zero-area boxes, and label mismatches. To ensure annotation accuracy, strict quality control was applied to the converted labels. Manual verification confirmed that the bounding boxes completely cover the targets to minimize noise. As illustrated in Figure 6, the dataset encompasses 17 biological categories and details the per-category image distribution.

**Figure 7:**
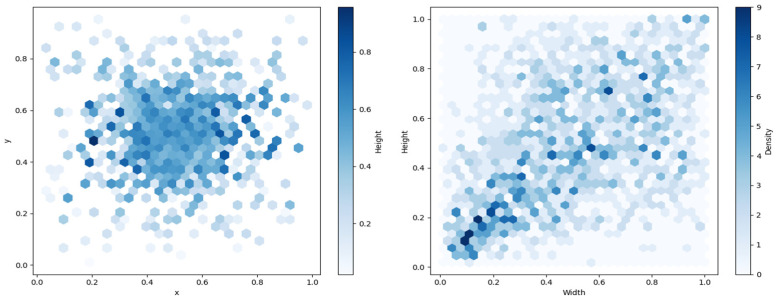
Distribution of Ground Truth Box Center Coordinates, Width, and Height.

The UCOD10K dataset trains models by incorporating unlabeled background images to improve the model’s ability to identify subtle camouflaged objects. The distributions of ground truth box coordinates and target box dimensions are illustrated in Figure 7.

The center points of the boxes are evenly distributed, which means that camouflaged objects appear throughout the image. This balanced distribution is beneficial for training models, ensuring that the model can detect objects in various regions of the image. At the same time, the sizes of the ground truth boxes are widely distributed, presenting a rigorous test for the model’s ability to handle extreme scale variations.

**Figure 8:**
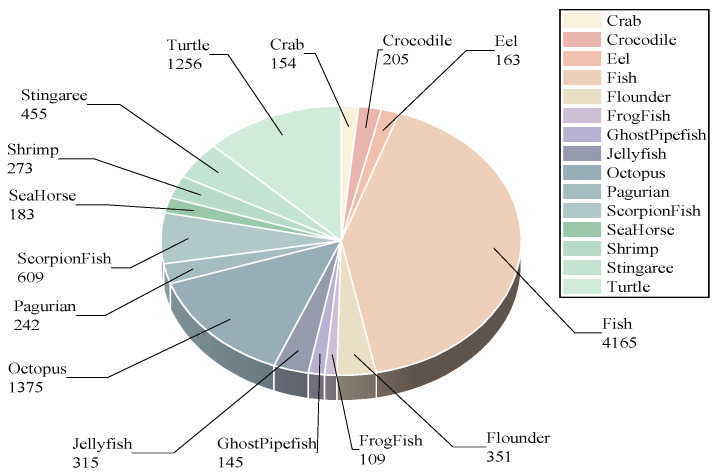
Category Distribution of the UWB-COT220 Dataset.

In addition, to validate the robustness of our method under diverse camouflage patterns and imaging conditions, we introduce the UWB-COT220 dataset. It consists of 10,000 images sampled from the UW-COT220 video collection [14], and its annotation protocol is consistent with UCOD10K, covering a 15-category subset. The category distribution of UWB-COT220 is given in Figure 8. Both datasets feature typical perceptual challenges of underwater camouflage, including low light, water turbidity, target occlusion, and low foreground-to-background contrast.

#### 4.1.3. Evaluation Metrics

To comprehensively evaluate CAR-YOLO, we adopt standard object detection metrics, including precision (P), recall (R), mAP50, and COCO-style mAP50-95, following the widely used PASCAL VOC [33] and MS COCO [34] evaluation protocols. Model complexity is measured in terms of the total number of parameters and GFLOPs.

Unless otherwise specified, all training and ablation experiments are conducted on the UCOD10K dataset, which is split into training, validation, and test sets with a 6:2:2 ratio. To further validate model performance, we test on the UWB-COT220 dataset, which consists of 10,000 video-derived underwater images and is divided into 6:2:2 subsets using the same protocol.

**Figure 9:**
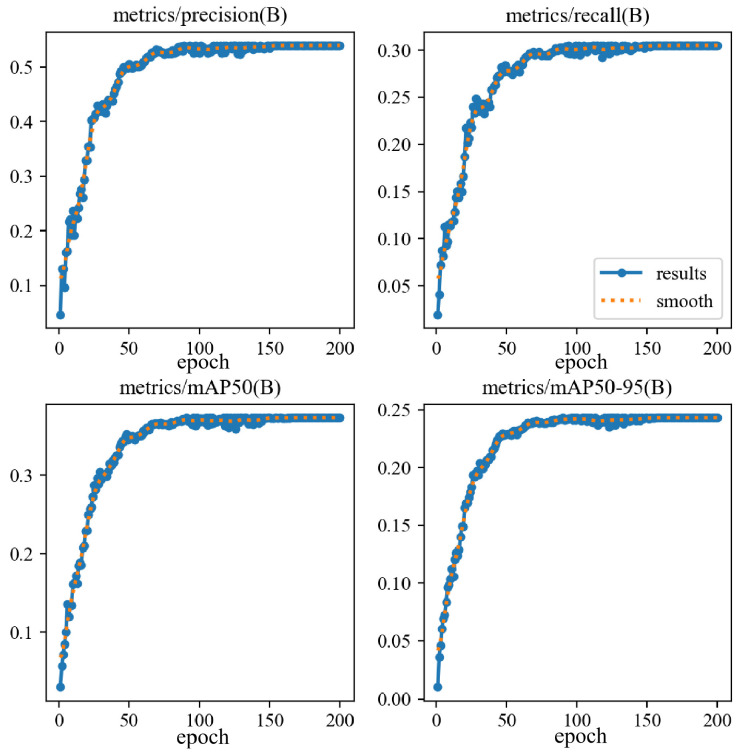
Training curves of CAR-YOLO on the UCOD10K dataset.

The CAR-YOLO model is trained for 200 epochs on UCOD10K with fixed data splits and a random seed. During training, the total loss decreases rapidly and then gradually converges, while P, R, mAP50, and mAP50-95 continuously improve and eventually stabilize. The results of CAR-YOLO trained for 200 epochs on the UCOD10K dataset are shown in Figure 9.

**Figure 10:**
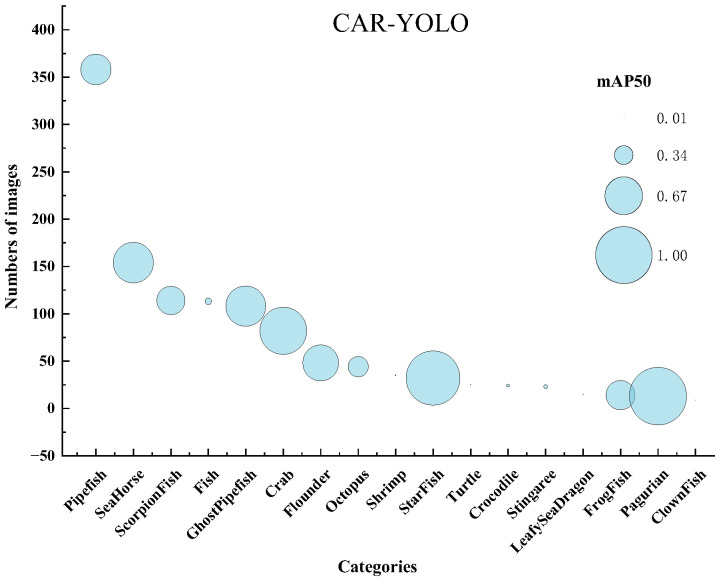
Category-wise mAP50 on the UCOD10K dataset.

As shown in Figure 9, the overall detection accuracy of CAR-YOLO is relatively modest. The reason is mainly due not to insufficient training, but to the intrinsically long-tailed nature of the dataset. Common species such as *Pipefish* and *Sea Horse* contain hundreds of annotated images, whereas rare species like *Leafy Sea Dragon* and *Clown Fish* appear only a few times in the original COD10K annotations. Moreover, underwater camouflaged object detection is inherently challenging because of complex backgrounds and low target visibility. The number of images per category and the corresponding mAP50 values are visualized in Figure 10, where the x-axis denotes the object categories, the y-axis indicates the number of annotated images per category, and the bubble size encodes the per-class mAP50.

To mitigate the adverse effects of rare classes in the long-tailed distribution, we utilized Mosaic data augmentation to increase the diversity of rare classes and leveraged the Focal Loss mechanism. Focal Loss mechanism automatically down-weights easy examples and focuses training gradients on hard, misclassified samples. This ensures that the model prioritizes under-represented categories without requiring manual weight adjustments. CAR-YOLO achieves relatively high and stable mAP50 scores for head and medium-frequency categories, which contain sufficient training samples. This performance demonstrates that the model effectively handles these well-represented classes.

### 4.2. Ablation Study

For the purpose of validating the effectiveness of the FRM-DWT, EG-IWT, and the various implicit calibration components within UCH, we conducted a series of exhaustive ablation studies on the UCOD10K dataset, using YOLOv11n as the baseline. All modules were added incrementally to evaluate their marginal contribution, as shown in Table 2. “√” indicates that we used this module.

Table 2 demonstrates the individual contributions and synergistic effects of each module. Compared to the YOLOv11 baseline, incorporating all modules significantly improves Precision, Recall, and mAP metrics.

It can be seen that UCH yields the most significant performance gains. Operating as a zero-overhead supervision strategy, it boosts Precision by 6.1% and mAP50-95 by 1.4%. This gain stems from its ability to calibrate noisy supervision signals using uncertainty modeling and consistency guidance, rather than adding heavy feature extraction layers.

Frequency-domain modules play pivotal roles. FRM-DWT independently increases Precision by 4.4%, highlighting the critical value of preserving high-frequency boundary information during downsampling. EG-IWT raises mAP50-95 by 0.8%. The result confirms that dynamically restoring high-frequency contours outperforms simple interpolation.

The LAF module optimizes the balance between performance and efficiency. Beyond a 0.7% mAP50-95 improvement, its primary contribution lies in model compression, reducing parameters from 2.58 M to 2.35 M and GFLOPs from 6.3 to 5.5. This efficiency demonstrates that LAF performs semantic fusion more effectively than standard concatenation.

**Figure 11:**
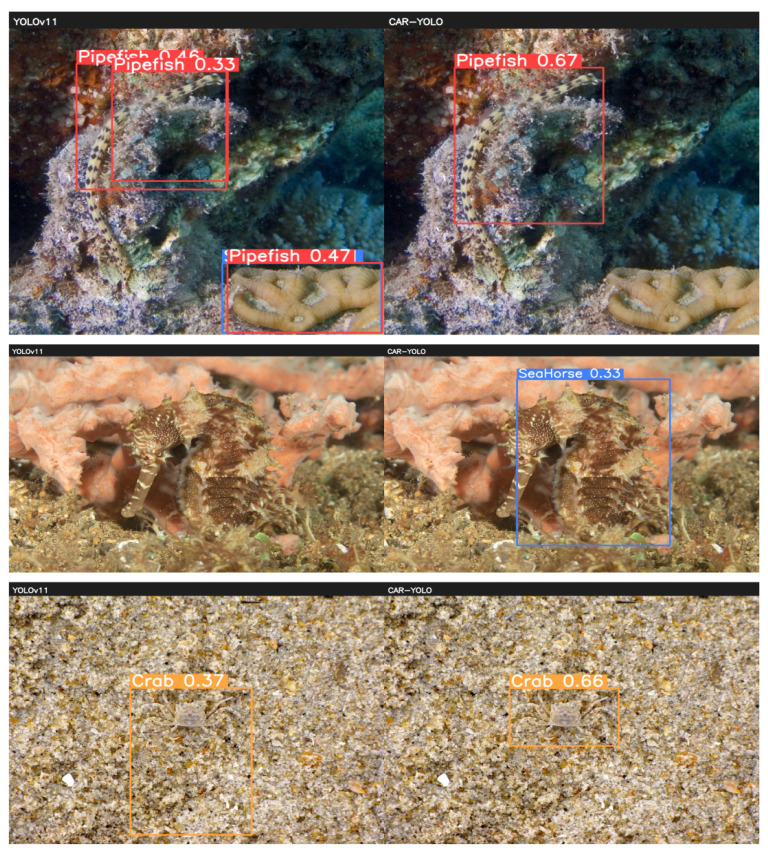
Comparison of YOLOv11 and CAR-YOLO Inference Results.

When these modules are combined, their synergistic effects are further enhanced. The combination of FRM-DWT and LAF not only improves Precision and mAP50-95 but also maintains a smaller model and higher efficiency, fully demonstrating that effective fusion amplifies the benefits of frequency-domain downsampling. Similarly, combining LAF and UCH achieves high precision with low complexity. Overall, the composite of these modules significantly improves the model’s detection accuracy while reducing computational complexity and parameter count, confirming the effectiveness of CAR-YOLO in enhancing model performance at multiple levels, as shown in Figure 11.

Finally, CAR-YOLO achieved an mAP50-95 of 0.271 and a Precision of 0.743, surpassing the baseline by 2.8% and 30.3%, respectively. Despite these gains, it maintains lower computational costs with only 2.43 M parameters and 5.9 GFLOPs. To validate reliability, we trained the final model five times, obtaining an average mAP of 40.5% with a standard deviation of 0.23%. This minimal variance confirms that the performance improvements are statistically significant and not a result of random noise.

### 4.3. Comparative Experiment

To further demonstrate the advantages, we compared with other common general-purpose object detectors on the UCOD10K and UWB-COT220 datasets, including YOLOv5 [35] through YOLOv10 [36] and YOLOv11 [22] series, as well as two underwater detection methods, LFN-YOLO [20] and Dynamic YOLO [21]. The results are shown in Table 3 and in Table 4.

On the UCOD10K dataset, CAR-YOLO achieves the best performance across all metrics. Its lightweight is particularly evident when compared to recent SOTA variants. Dynamic YOLO requires 8.27 M parameters and 12.56 GFlops. LFN-YOLO utilizes 2.7 M parameters and 7.2 GFlops. In contrast, CAR-YOLO operates with only 2.43 M parameters and 5.9 GFlops. The inference time for each image is 1.2 ms. This demonstrates that CAR-YOLO significantly reduces computational cost while maintaining superior detection accuracy. Calculated via Precision and Recall, the F1-score of CAR-YOLO is 0.496. Compared to the baseline YOLOv11n, our model improves mAP50-95 by 11.5% and achieves the highest F1-score among all compared methods.

**Figure 12:**
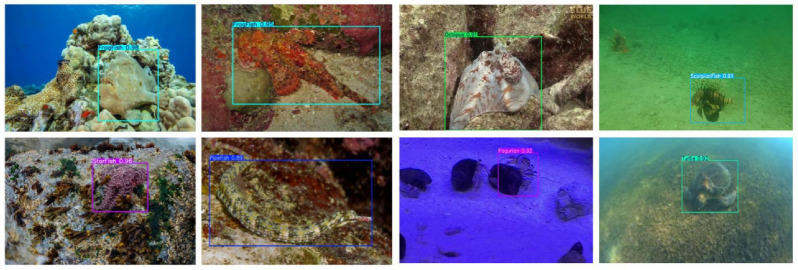
Detection examples of CAR-YOLO on UCOD10k and UWB-COT220.

CAR-YOLO’s advantages were further validated on the UWB-COT220 dataset. This dataset has a considerable number of frames, and the challenges of low contrast and blurry boundaries are more severe. In this more difficult setting, CAR-YOLO’s accuracy improvement was also significant. Its mAP50-95 reached 0.307, achieving a 33.5% substantial improvement over YOLOv11, and its Recall also increased significantly by 12.97%. Figure 12 shows some inference results of CAR-YOLO on the UCOD10K and UWB-COT220 test set.

The aforementioned results indicates that the model has the potential to achieve high accuracy under optimal conditions. This highlights the progress made in underwater object detection. Similarly, various underwater targets such as frogfish, scorpionfish, and flounders can be seen. CAR-YOLO can identify these targets in complex underwater environments.

**Figure 13:**
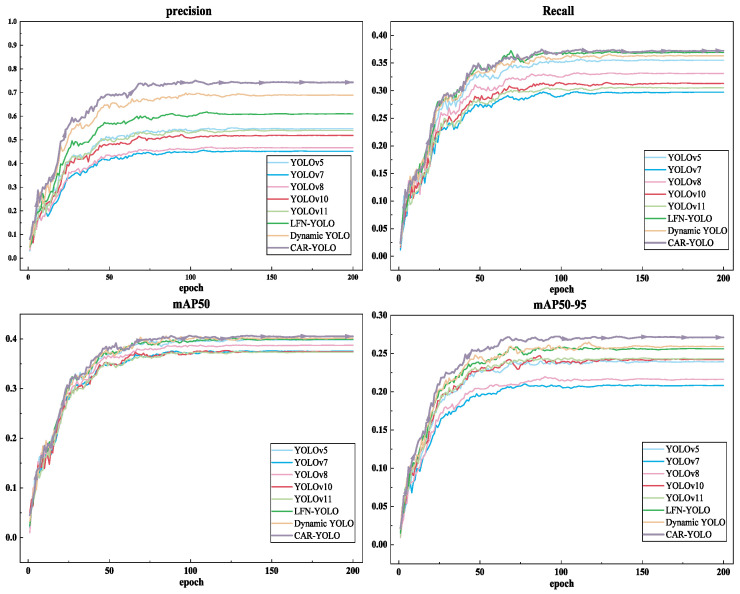
Evaluation curves of all compared models on the UCOD10K dataset.

Additionally, Figure 13 plots the evaluation curves for all compared models on the UCOD10K dataset, covering Precision, Recall, mAP50, and mAP50-95.

Compared to the baselines, CAR-YOLO exhibits much smoother training curves with fewer fluctuations. This indicates a more stable learning process across the 200 epochs.

In summary, CAR-YOLO’s performance is primarily attributed to its superior localization quality. Across both datasets, the improvement margin for mAP50-95 consistently exceeds that of mAP50. This confirms the efficiency of our modules in preserving and leveraging high-frequency boundary information, which significantly improves localization regression quality, especially at higher IoU thresholds.

**Figure 14:**
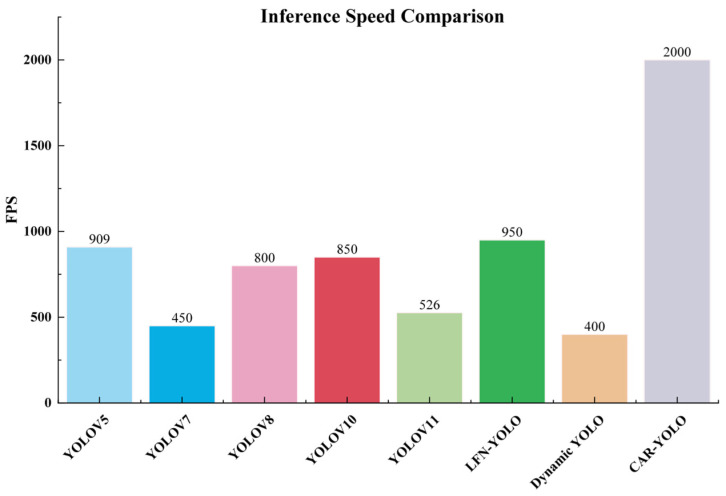
Comparative analysis of inference speed (FPS) across different detection models.

To rigorously validate the deployment feasibility of CAR-YOLO, we conducted a systematic runtime evaluation under a controlled hardware environment to assess its real-time processing capabilities, such as Figure 14.

All evaluations were conducted on an NVIDIA GeForce RTX 4090 GPU with a batch size of eight and FP16 precision. As shown in Figure 14, the proposed CAR-YOLO achieves a remarkable inference speed of 2000 FPS, surpassing the baseline YOLOv11 (526 FPS) by approximately four times and significantly outperforming other state-of-the-art lightweight detectors. Crucially, CAR-YOLO achieves superior accuracy alongside a highly lightweight architecture. By employing parameter-efficient low-rank structures in LAF and the zero-overhead UCH strategy, we minimize computational costs, outperforming existing state-of-the-art lightweight detectors in efficiency. This design proves that frequency-tailored networks can offer high practical value. They are ideal for real-time deployment on resource-constrained underwater platforms.

### 4.4. Visualization and Interpretability Analysis

**Figure 15:**
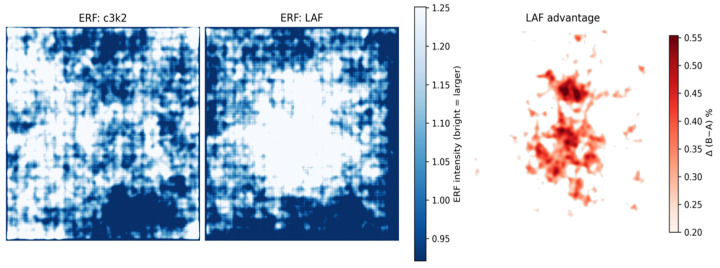
Effective Receptive Field (ERF) visualization of the LAF module.

With the aim of further analyzing the LAF module, we calculated its Effective Receptive Field (ERF). The method we adopted is based on gradient backpropagation:

Specifically, we selected the target layer where the LAF module is located, as well as the corresponding C3k2 module that was replaced, and calculated the average energy of its output feature map. This energy value was then used as the loss for backpropagation, traced all the way back to the original input image. The sum of the absolute values of the gradients accumulated on the input image constitutes the Effective Receptive Field heatmap for that layer. As shown in Figure 15, this map visually quantifies the actual contribution or influence of each input pixel on that deep feature map.

Figure 15 compares the Effective Receptive Field of the standard C3k2 module and our LAF module. The C3k2 baseline exhibits a restricted and scattered receptive field. In contrast, the LAF module shows a significantly expanded high-response region. The center becomes very dense and bright, covering most of the central area. This expanded and concentrated field of view allows the model to capture broader context, which is critical for recognizing camouflaged objects.

**Figure 16:**
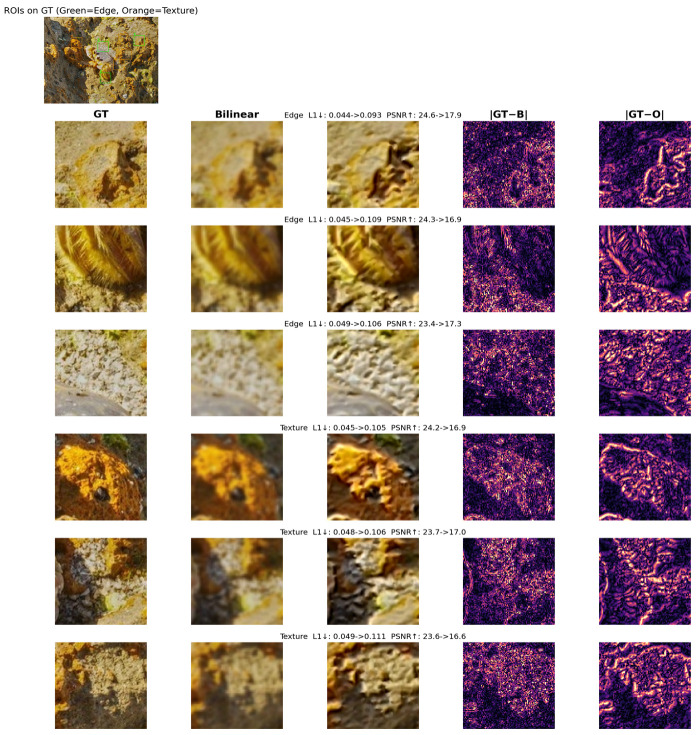
Difference heatmap between EG-IWT and Bilinear Interpolation upsampling. “ ->” indicates value change; “↓”/“↑” denote lower/higher is better; colored boxes highlight zoomed-in regions.

To further confirm that the EG-IWT module can effectively extract high-frequency boundary features from feature maps, we adopted the method illustrated in Figure 16 to obtain and compare the difference heatmaps between the traditional bilinear interpolation upsampling module and the EG-IWT module.

As shown in Figure 16, traditional bilinear interpolation causes significant blurring and detail loss. In contrast, EG-IWT effectively recovers these details, producing sharper features closer to the original image.

The difference maps in the last two columns confirm this improvement. The error map for bilinear interpolation ($\left|GT-B\right|$) exhibits widespread bright noise and artifacts, indicating high reconstruction errors across the region. In comparison, the EG-IWT map ($\left|GT-O\right|$) appears significantly darker and cleaner. It suppresses most noise, demonstrating EG-IWT’s superiority in preserving high-frequency information and texture details.

In deep networks, gradients naturally weaken during backpropagation from the detection head to the backbone. In YOLOv11, the gradient strength diminishes significantly after C2 and becomes weak by C3. This attenuation leaves shallow layers under-supervised, hindering the learning of fine boundaries and textures. To address this, the UCH module calibrates supervision in ambiguous regions and redistributes gradients toward informative boundary cues.

**Figure 17:**
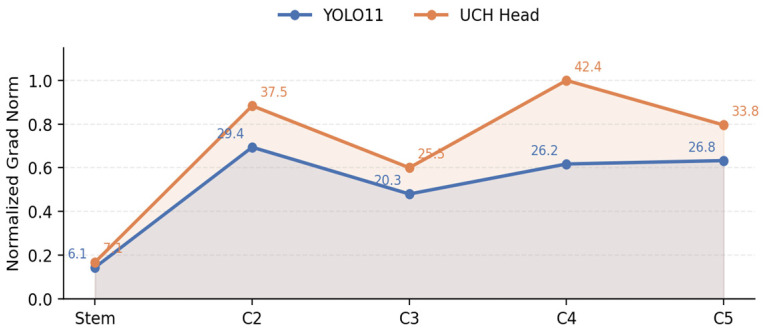
Visualization of Gradient Attenuation Mitigation by UCH.

For a quantitative assessment of this improvement, we analyzed the gradient flow. Backward hooks were registered at five key backbone stages, corresponding to downsampling strides of 2, 4, 8, 16, and 32. Following forward and backward propagation, we extracted the output gradients. Subsequently, we computed and normalized their average L2 norms. Figure 17 visualizes the resulting curves.

As illustrated in Figure 17, the original YOLOv11 head exhibits pronounced gradient attenuation, with the gradient magnitude dropping sharply after C2. In contrast, the model equipped with UCH consistently yields higher gradient norms at the C2, C3, and C4 stages, indicating stronger training signals propagating to the shallow and middle layers. This mitigates gradient attenuation along the backbone and enables a more uniform and sufficient optimization of the entire network.

**Figure 18:**
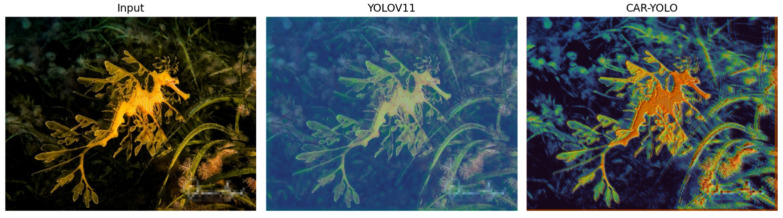
Heatmap Comparison of Camouflaged Target Perception.

In an effort to intuitively compare the models’ perception capabilities, we visualized the output of the highest-resolution pyramid feature layer (P3). Activation maps were averaged across the channel dimension to generate single-channel heatmaps. Since the P3 features have a lower resolution, we upsampled these heatmaps to the original image size and overlaid them onto the input images. This superimposition allows for a clear visualization of the focus areas against the object details. To ensure a fair comparison, the heatmaps for both models were processed using uniform quantile normalization and the same pseudo-color mapping. The resulting comparison is shown in Figure 18.

YOLOv11 exhibits a weak and scattered response to the *LeafySeaDragon*. Its heatmap lacks definition along the target’s leaf-like appendages and edges. In contrast, CAR-YOLO utilizes wavelet-based modules to solve this. FRM-DWT and EG-IWT explicitly preserve and reconstruct high-frequency details during sampling, avoiding the blurring inherent in standard methods. The LAF module further enhances semantic features and boundary continuity. Subsequently, the UCH refines these optimized features for precise localization. Consequently, CAR-YOLO demonstrates superior focus. The heatmap clearly delineates the target with comprehensive coverage. It effectively suppresses background noise and eliminates false detections.

### 4.5. Generalization Experiments

**Figure 19:**
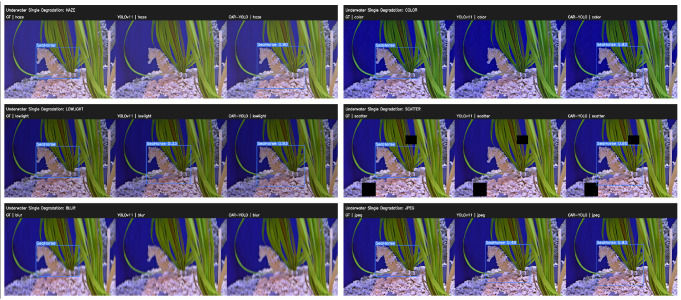
Qualitative comparison between YOLOv11 and CAR-YOLO under various underwater degradation scenarios. The black blocks indicate occluded regions.

We designed a set of experiments to evaluate the CAR-YOLO model’s generalization performance in complex underwater environments. Six independent tests were made by systematically applying simulated visual corruptions to the baseline images. As in Figure 19, these common underwater degradations include haze, color distortion, low brightness, occlusion, blur, and compression artifacts. Subsequently, we evaluated the detection performance of both the YOLOv11 and CAR-YOLO.

Qualitative results show that CAR-YOLO outperforms YOLOv11n across most degradation scenarios. In the SCATTER (occlusion) test, YOLOv11n misses the target entirely, while CAR-YOLO achieves a detection confidence of 0.95. Although both models fail in extreme Blur cases due to severe contrast loss, these represent common physical limits. Overall, CAR-YOLO remains more reliable than the baseline. It provides stable detection in challenging underwater conditions where standard models fail. We further evaluate it on the UODD benchmark [37] to verify CAR-YOLO’s generalization capability on generic underwater object detection. UODD originates from real-world coastal aquaculture scenes. It contains 3194 images with 19,212 annotated instances across three categories: scallop, sea cucumber, and sea urchin. We split the dataset into 2560 training images, 128 validation images, and 506 test images.

**Figure 20:**
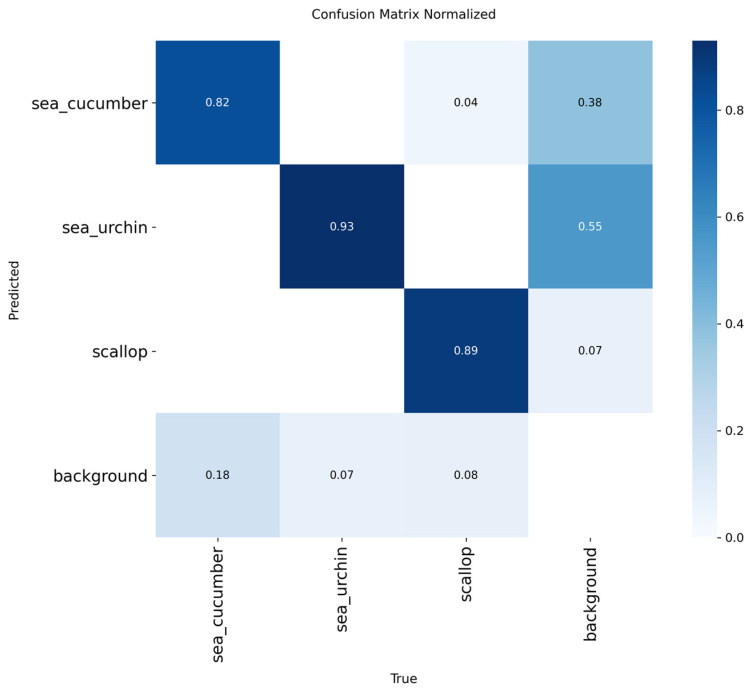
Normalized confusion matrix of CAR-YOLO on the UODD dataset.

To analyze the specific class-wise performance of CAR-YOLO, Figure 20 presents the normalized confusion matrix generated on the UODD dataset. First, the model achieves high recognition rates for distinctive targets, with 93% for sea urchins and 89% for scallops. More importantly, for sea cucumbers, which are highly camouflaged and blend seamlessly into the seabed, CAR-YOLO still maintains a robust accuracy of 82%. This demonstrates that our frequency-domain and fusion designs effectively extract subtle boundary cues, allowing the model to overcome strong background interference. Overall, the matrix confirms that CAR-YOLO is reliable for both general and camouflaged underwater species.

In addition, we include DJL-Net [38], a DINO-based underwater detector that achieves strong performance on UODD as well as on RUOD and UDD. The comparison results on the UODD test set are shown in Table 5.

The quantitative results on the UODD dataset are shown in Table 5. CAR-YOLO achieves a mAP50 of 90.1%, while YOLOv11n and DJL-Net reach 88.3% and 88.8%, respectively. Regarding mAP50-95, our model scores 53.8%, outperforming the 53.0% achieved by the YOLOv11n baseline. Furthermore, CAR-YOLO is significantly more efficient. It uses only 2.43 M parameters and 5.9 GFlops, whereas the heavyweight DJL-Net requires 69.5 M parameters and 58.5 GFlops.

**Figure 21:**
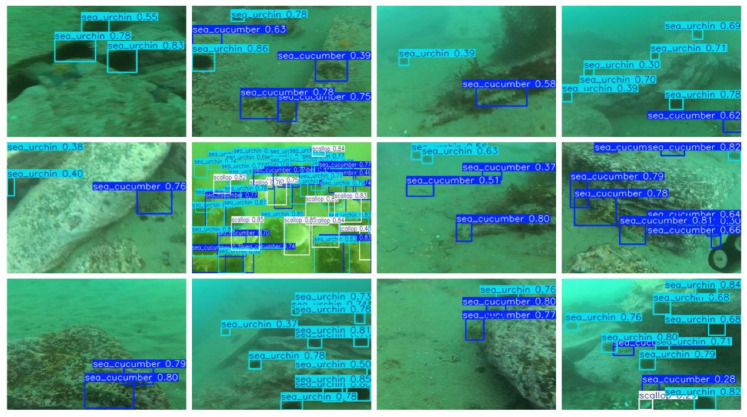
Detection examples of CAR-YOLO on UODD.

The inference results of CAR-YOLO on the UODD dataset are in Figure 21. The UODD dataset is characterized by underwater image degradation and a significant number of small targets, making it challenging for detection models. Despite these difficulties, better performance is shown in identifying and localizing small objects precisely.

More importantly, CAR-YOLO holds a significant advantage in terms of model efficiency. It reduces the parameter count to 2.43 million. This is drastically lower than the 69.5 million parameters required by DJL-Net. These figures confirm that CAR-YOLO successfully balances high generalization capability with extreme lightweight efficiency. Our model with an inference time of 1.1ms per image on this dataset. These three metrics are the lowest among all compared models, which facilitates CAR-YOLO deployment on edge devices, such as ROVs and USVs.

## 5. Conclusions

This paper proposes CAR-YOLO, a lightweight framework designed to solve the boundary ambiguity challenge in UCOD. It integrates three core innovations: the frequency-domain mechanisms (FRM-DWT and EG-IWT), the LAF dynamic fusion module, and the UCH uncertainty calibration head. Extensive experiments on the UCOD10K and UWB-COT220 benchmarks confirm the model’s effectiveness.

Despite these achievements, the current framework exhibits limitations under extreme degradation scenarios. Primarily, the reliance on wavelet-based enhancement restricts performance in severely turbid or highly blurred conditions, where high-frequency cues are physically obliterated. Similarly, the model faces challenges in extremely low-light environments due to drastic contrast reduction, as well as with heavily occluded small objects where semantic information is scarce. In future work, we plan to address these physical constraints by exploring multi-modal fusion strategies using sonar or depth data. We also aim to integrate video attention mechanisms to leverage temporal consistency. Ultimately, we aim to deploy the optimized model on resource-constrained platforms, such as USVs and ROVs, to ensure real-time underwater monitoring in practical applications.

## Figures and Tables

**Table 1:** Training Parameters.

Parameter	Value
Image Input Size	640 × 640
Batch Size	8
Optimizer	Stochastic Gradient Descent (SGD)
Initial Learning Rate	5.0 × 10^−4^
Weight Decay	5.0 × 10^−4^
Warm-up Epochs	5
Stabilization Epochs	60
Epochs	200

**Table 2:** Ablation experiments on UCOD10K.

Module				P	R	mAP50	mAP50-95	Params (M)	GFlops
LAF	FRM-DWT	EG-IWT	UCH						
				0.54	0.305	0.373	0.243	2.58	6.3
√				0.543	0.311	0.374	0.25	2.35	5.5
	√			0.584	0.342	0.377	0.253	2.59	6.3
		√		0.571	0.335	0.369	0.251	2.6	6.4
			√	0.601	0.352	0.38	0.257	2.58	6.3
√	√	√		0.652	0.359	0.394	0.261	2.38	5.7
√			√	0.709	0.366	0.397	0.264	2.35	5.7
	√	√	√	0.711	0.368	0.4	0.268	2.61	6.4
√	√	√	√	0.743	0.372	0.405	0.271	2.43	5.9

**Table 3:** Performance comparison of CAR-YOLO against SOTA methods on the UCOD10K set.

Arithmetic	P	R	mAP50	mAP50-95	Params(M)	GFlops
YOLOV5n	0.547	0.355	0.398	0.239	2.50	7.1
YOLOV7n	0.452	0.297	0.375	0.208	6.0	13.2
YOLOV8n	0.467	0.331	0.387	0.216	3.01	8.1
YOLOV10n	0.519	0.313	0.374	0.242	2.7	8.2
YOLOV11n	0.54	0.305	0.373	0.243	2.58	6.3
LFN-YOLO	0.610	0.369	0.399	0.256	2.7	7.2
Dynamic YOLO	0.689	0.363	0.401	0.259	8.27	12.56
CAR-YOLO	0.743	0.372	0.405	0.271	2.43	5.9

**Table 4:** Performance comparison of CAR-YOLO against SOTA methods on the UWB-COT220 set.

Arithmetic	P	R	mAP50	mAP50-95	Params (M)	GFlops
YOLOV5n	0.508	0.312	0.358	0.207	2.50	7.1
YOLOV7n	0.493	0.3	0.339	0.198	6.0	13.2
YOLOV8n	0.531	0.325	0.341	0.201	3.01	8.1
YOLOV10n	0.543	0.334	0.359	0.206	2.7	8.2
YOLOV11n	0.61	0.347	0.361	0.23	2.58	6.3
LFN-YOLO	0.605	0.354	0.381	0.271	2.7	7.2
Dynamic YOLO	0.611	0.378	0.384	0.289	8.27	12.56
CAR-YOLO	0.618	0.392	0.393	0.307	2.43	5.9

**Table 5:** Performance comparison of CAR-YOLO against SOTA methods on the UODD set.

Arithmetic	P	R	mAP50	mAP50-95	Params (M)	GFlops
YOLOV11n	0.85	0.839	0.883	0.53	2.58	6.3
DJL-Net	0.873	0.819	0.888	0.529	69.5	58.5
CAR-YOLO	0.885	0.829	0.901	0.538	2.43	5.9

## Data Availability

The data presented in this study are available on request from the corresponding author.

## References

[1] Fan D.-P., Ji G.-P., Sun G., Cheng M.-M., Shen J., Shao L. Camouflaged Object Detection. Proceedings of the IEEE/CVF Conference on Computer Vision and Pattern Recognition (CVPR).

[2] He C., Li K., Zhang Y., Tang L., Zhang Y., Guo Z., Li X. Camouflaged Object Detection with Feature Decomposition and Edge Reconstruction. Proceedings of the IEEE/CVF Conference on Computer Vision and Pattern Recognition (CVPR).

[3] Chen L., Huang Y., Dong J., Xu Q., Kwong S., Lu H., Lu H., Li C. (2025). Underwater Optical Object Detection in the Era of Artificial Intelligence: Current, Challenge, and Future. ACM Comput. Surv..

[4] Bi H., Zhang C., Wang K., Tong J., Zheng F. (2022). Rethinking Camouflaged Object Detection: Models and Datasets. IEEE Trans. Circuits Syst. Video Technol..

[5] Jiao P., Ye X., Zhang C., Li W., Wang H. (2024). Vision-Based Real-Time Marine and Offshore Structural Health Monitoring System Using Underwater Robots. Comput.-Aided Civ. Infrastruct. Eng..

[6] Yang X., Shao S., Liu W., Xiang Z., Zhang B. (2025). TAD-YOLO: A Lightweight Nearshore Ship Detection Method for Small USVs in Maritime Trade Surveillance. Ocean. Eng..

[7] Yang X., She H., Lou M., Ye H., Guan J., Li J., Xiang Z., Shen H., Zhang B. (2025). A Joint Ship Detection and Waterway Segmentation Method for Environment-Aware of USVs in Canal Waterways. IEEE Trans. Autom. Sci. Eng..

[8] Redmon J., Divvala S., Girshick R., Farhadi A. You Only Look Once: Unified, Real-Time Object Detection. Proceedings of the IEEE Conference on Computer Vision and Pattern Recognition (CVPR).

[9] Huang Z., Dai H., Xiang T.-Z., Wang S., Chen H.-X., Qin J., Xiong H. Feature Shrinkage Pyramid for Camouflaged Object Detection with Transformers. Proceedings of the IEEE/CVF Conference on Computer Vision and Pattern Recognition (CVPR).

[10] Wang T., Yu Z., Fang J., Xie J., Yang F. (2025). Multidimensional Fusion of Frequency and Spatial Domain Information for Enhanced Camouflaged Object Detection. Inf. Fusion.

[11] Zhao X., Ming Q., Yang Y., Hu W., Li W., Tao R. (2025). Chirplet Fourier Analysis Network for Cross-Scene Classification of Multisource Remote Sensing Data. IEEE Trans. Geosci. Remote Sens..

[12] Zhang P., Yan T., Liu Y., Lu H. Fantastic Animals and Where to Find Them: Segment Any Marine Animal with Dual SAM. Proceedings of the IEEE/CVF Conference on Computer Vision and Pattern Recognition (CVPR).

[13] Ke L., Ye M., Danelljan M., Liu Y., Tai Y.-W., Tang C.-K., Yu F., Oh A., Naumann T., Globerson A., Saenko K., Hardt M., Levine S. (2023). Segment Anything in High Quality. Advances in Neural Information Processing Systems.

[14] Zhang C., Liu L., Huang G., Zhang Z., Wen H., Zhou X., Ge S., Wang Y. Underwater Camouflaged Object Tracking Meets Vision-Language SAM2. Proceedings of the IEEE/CVF Conference on Computer Vision and Pattern Recognition (CVPR) Workshops.

[15] Zhang H., Li F., Liu S., Zhang L., Su H., Zhu J., Ni L.M., Shum H.-Y. DINO: DETR with Improved DeNoising Anchor Boxes for End-to-End Object Detection. Proceedings of the International Conference on Learning Representations (ICLR).

[16] Cao R., Zhang R., Yan X., Zhang J. (2024). BG-YOLO: A Bidirectional-Guided Method for Underwater Object Detection. Sensors.

[17] Liu K., Peng L., Tang S. (2023). Underwater Object Detection Using TC-YOLO with Attention Mechanisms. Sensors.

[18] Yeh C.-H., Lin C.-H., Kang L.-W., Huang C.-H., Lin M.-H., Chang C.-Y., Wang C.-C. (2022). Lightweight Deep Neural Network for Joint Learning of Underwater Object Detection and Color Conversion. IEEE Trans. Neural Netw. Learn. Syst..

[19] Jiang L., Wang Y., Jia Q., Xu S., Liu Y., Fan X., Li H., Liu R., Xue X., Wang R. Underwater Species Detection Using Channel Sharpening Attention. Proceedings of the 29th ACM International Conference on Multimedia (MM ‘21).

[20] Liu M., Wu Y., Li R., Lin C. (2025). LFN-YOLO: Precision Underwater Small Object Detection via a Lightweight Reparameterized Approach. Front. Mar. Sci..

[21] Chen J., Er M.J. (2024). Dynamic YOLO for Small Underwater Object Detection. Artif. Intell. Rev..

[22] Khanam R., Hussain M. (2024). YOLO11: An Overview of the Key Architectural Enhancements. arXiv.

[23] Zunair H., Khan S., Hamza A.B. RSUD20K: A Dataset for Road Scene Understanding in Autonomous Driving. Proceedings of the 2024 IEEE International Conference on Image Processing (ICIP).

[24] Liu Z., Deng X., Jiang P., Lv C., Min G., Wang X. (2024). Edge Perception Camouflaged Object Detection Under Frequency Domain Reconstruction. IEEE Trans. Circuits Syst. Video Technol..

[25] Hu E.J., Shen Y., Wallis P., Allen-Zhu Z., Li Y., Wang S., Wang L., Chen W. LoRA: Low-Rank Adaptation of Large Language Models. Proceedings of the International Conference on Learning Representations (ICLR).

[26] Yang L., Zhang R.-Y., Li L., Xie X. SimAM: A Simple, Parameter-Free Attention Module for Convolutional Neural Networks. Proceedings of the 38th International Conference on Machine Learning (ICML).

[27] Ding X., Zhang X., Han J., Ding G. Scaling Up Your Kernels to 31x31: Revisiting Large Kernel Design in CNNs. Proceedings of the IEEE/CVF Conference on Computer Vision and Pattern Recognition (CVPR).

[28] Ming Q., Miao L., Zhou Z., Vercheval N., Pizurica A. (2024). Not All Boxes Are Equal: Learning to Optimize Bounding Boxes With Discriminative Distributions in Optical Remote Sensing Images. IEEE Trans. Geosci. Remote Sens..

[29] Ming Q., Miao L., Zhou Z., Song J., Pizurica A. (2024). Gradient Calibration Loss for Fast and Accurate Oriented Bounding Box Regression. IEEE Trans. Geosci. Remote Sens..

[30] Zhang H., Xu C., Zhang S. (2023). Inner-IoU: More Effective Intersection over Union Loss with Auxiliary Bounding Box. arXiv.

[31] Rezatofighi H., Tsoi N., Gwak J., Sadeghian A., Reid I., Savarese S. Generalized Intersection Over Union: A Metric and a Loss for Bounding Box Regression. Proceedings of the IEEE/CVF Conference on Computer Vision and Pattern Recognition (CVPR).

[32] Li X., Wang W., Wu L., Chen S., Hu X., Li J., Tang J., Yang J., Larochelle H., Ranzato M., Hadsell R., Balcan M.F., Lin H. (2020). Generalized Focal Loss: Learning Qualified and Distributed Bounding Boxes for Dense Object Detection. Advances in Neural Information Processing Systems.

[33] Everingham M., Van Gool L., Williams C.K.I., Winn J., Zisserman A. (2010). The Pascal Visual Object Classes (VOC) Challenge. Int. J. Comput. Vis..

[34] Lin T.-Y., Maire M., Belongie S., Hays J., Perona P., Ramanan D., Dollár P., Zitnick C.L., Fleet D., Pajdla T., Schiele B., Tuytelaars T. (2014). Microsoft COCO: Common Objects in Context. Computer Vision—ECCV 2014.

[35] Terven J., Cordova-Esparza D. (2023). A comprehensive review of YOLO architectures in computer vision: From YOLOv1 to YOLOv8 and YOLO-NAS. Mach. Learn. Knowl. Extr..

[36] Sapkota R., Qureshi R., Calero M.F., Badjugar C., Nepal U., Poulose A., Vaddevolu U.B.P. (2024). YOLOv10 to its genesis: A decadal and comprehensive review of the You Only Look Once series. arXiv.

[37] Fu C., Fan X., Xiao J., Yuan W., Liu R., Luo Z. (2023). Learning Heavily-Degraded Prior for Underwater Object Detection. IEEE Trans. Circuits Syst. Video Technol..

[38] Wang B., Wang Z., Guo W., Wang Y. (2024). A Dual-Branch Joint Learning Network for Underwater Object Detection. Knowl.-Based Syst..

